# Supplementary material for: Improved in silico and in vitro methods for Escherichia coli LPS outer core typing
Source: Microb Genom. 2025 Sep 23;11(9):001506. doi: 10.1099/mgen.0.001506 (PMC13293308; doi:10.1099/mgen.0.001506)
Supplement: Uncited Supplementary Material 3. [file mgen-11-01506-s003.pdf]

Supplementary material

Improved *in silico* and *in vitro* methods for *E. coli* LPS outer core typing

Ellina Trofimova, Ruby P. Westerman, Paul R. Jaschke*

Author Affiliations:

School of Natural Sciences, Macquarie University, Sydney 2109, New South Wales, Australia ARC Centre of Excellence in Synthetic Biology, Macquarie University, Sydney, Australia

*Correspondence and requests for materials should be addressed to PRJ (paul.jaschke@mq.edu.au)

SUPPLEMENTARY FIGURES

Figure S1**.** Comparison of gene clusters between R2, K-12, R2/K-12, and K/12/R2 *E. coli* variants.

SUPPLEMENTARY TABLES

Table S21**.** LPS gene identity and similarity across K-12, R2 *E. coli* strains and their variants.

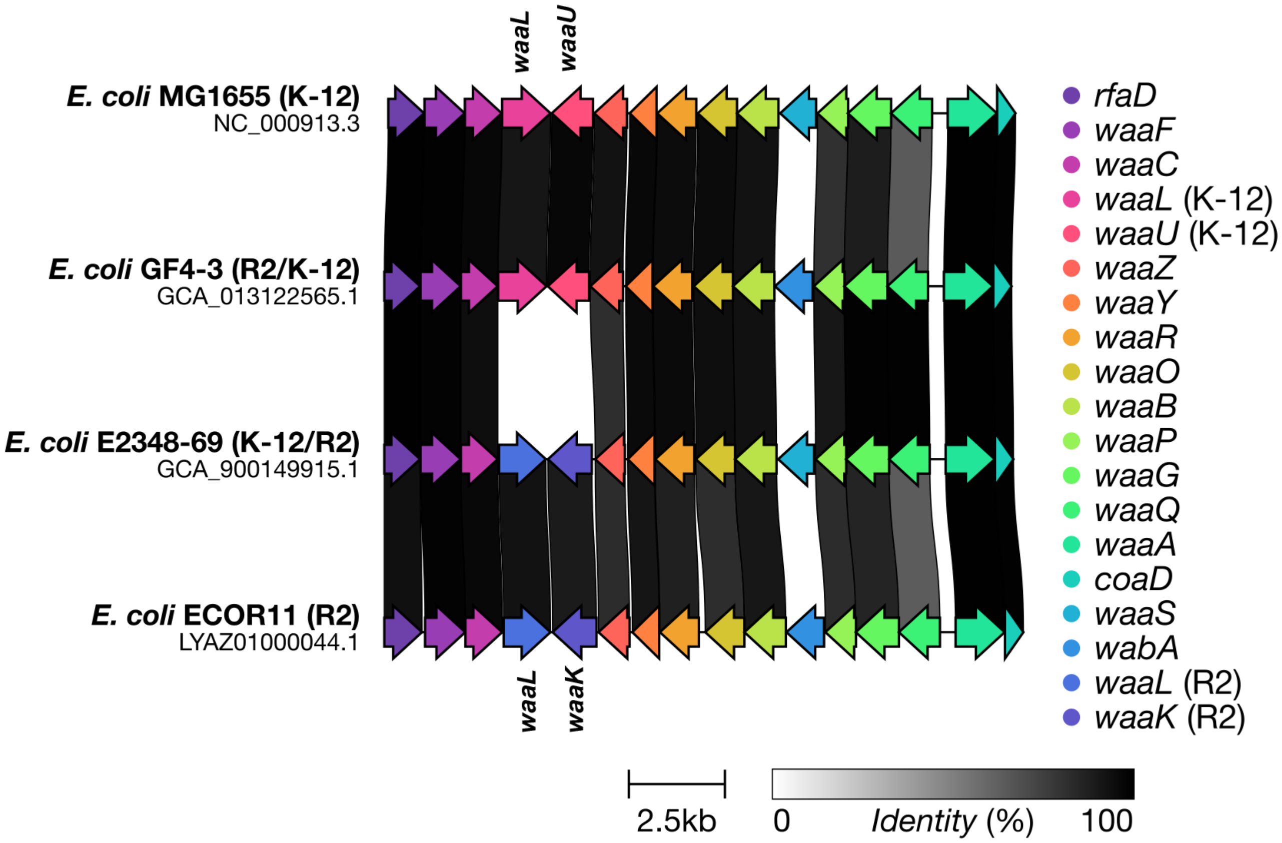

Figure S1. Comparison of gene clusters between R2, K-12, R2/K-12, and K/12/R2 *E. coli* variants. The comparison was performed using clinker [1]. R2/K-12 LPS outer core type designates R2 core with K-12 genes, and K-12/R2 – K-12 core with R2 genes. Numerical data is provided in Supplementary Table S21.

Table S21. LPS biosynthesis associated gene identity and similarity across K-12, R2 *E. coli* strains and their hybrids

***E. coli* strains alignment**	**Gene**	**Identity**	**Similarity**
E. coli MG1655 (K-12) vs *E.coli* GF4-3 (R2/K-12)	*rfaD*	1	1
E. coli MG1655 (K-12) vs *E.coli* GF4-3 (R2/K-12)	*waaF*	0.99	1
E. coli MG1655 (K-12) vs *E.coli* GF4-3 (R2/K-12)	*waaC*	0.98	0.98
E. coli MG1655 (K-12) vs *E.coli* GF4-3 (R2/K-12)	*waaL*	0.93	0.95
E. coli MG1655 (K-12) vs *E.coli* GF4-3 (R2/K-12)	*waaU*	0.97	0.99
E. coli MG1655 (K-12) vs *E.coli* GF4-3 (R2/K-12)	*waaZ*	0.94	0.96
E. coli MG1655 (K-12) vs *E.coli* GF4-3 (R2/K-12)	*waaY*	0.97	0.98
E. coli MG1655 (K-12) vs *E.coli* GF4-3 (R2/K-12)	*waaR*	0.96	0.98
E. coli MG1655 (K-12) vs *E.coli* GF4-3 (R2/K-12)	*waaO*	0.96	0.98
E. coli MG1655 (K-12) vs *E.coli* GF4-3 (R2/K-12)	*waaB*	0.94	0.96
E. coli MG1655 (K-12) vs *E.coli* GF4-3 (R2/K-12)	*waaP*	0.85	0.89
E. coli MG1655 (K-12) vs *E.coli* GF4-3 (R2/K-12)	*waaG*	0.9	0.94
E. coli MG1655 (K-12) vs *E.coli* GF4-3 (R2/K-12)	*waaQ*	0.72	0.81
E. coli MG1655 (K-12) vs *E.coli* GF4-3 (R2/K-12)	*waaA*	1	1
E. coli MG1655 (K-12) vs *E.coli* GF4-3 (R2/K-12)	*coaD*	0.99	0.99
E. coli MG1655 (K-12) vs *E. coli* isolate EPEC E2348_69 variety 2 (K-12/R2)	*rfaD*	1	1
E. coli MG1655 (K-12) vs *E. coli* isolate EPEC E2348_69 variety 2 (K-12/R2)	*waaF*	0.99	1
E. coli MG1655 (K-12) vs *E. coli* isolate EPEC E2348_69 variety 2 (K-12/R2)	*waaC*	0.95	0.97
E. coli MG1655 (K-12) vs *E. coli* isolate EPEC E2348_69 variety 2 (K-12/R2)	*waaZ*	0.85	0.87
E. coli MG1655 (K-12) vs *E. coli* isolate EPEC E2348_69 variety 2 (K-12/R2)	*waaY*	0.98	0.99
E. coli MG1655 (K-12) vs *E. coli* isolate EPEC E2348_69 variety 2 (K-12/R2)	*waaR*	0.96	0.99
E. coli MG1655 (K-12) vs *E. coli* isolate EPEC E2348_69 variety 2 (K-12/R2)	*waaO*	0.97	0.98

E. coli MG1655 (K-12) vs *E. coli* isolate EPEC E2348_69 variety 2 (K-12/R2)	*waaB*	0.93	0.96
E. coli MG1655 (K-12) vs *E. coli* isolate EPEC E2348_69 variety 2 (K-12/R2)	*waaS*	0.94	0.96
E. coli MG1655 (K-12) vs *E. coli* isolate EPEC E2348_69 variety 2 (K-12/R2)	*waaP*	0.9	0.94
E. coli MG1655 (K-12) vs *E. coli* isolate EPEC E2348_69 variety 2 (K-12/R2)	*waaG*	0.9	0.94
E. coli MG1655 (K-12) vs *E. coli* isolate EPEC E2348_69 variety 2 (K-12/R2)	*waaQ*	0.71	0.8
E. coli MG1655 (K-12) vs *E. coli* isolate EPEC E2348_69 variety 2 (K-12/R2)	*waaA*	1	1
E. coli MG1655 (K-12) vs *E. coli* isolate EPEC E2348_69 variety 2 (K-12/R2)	*coaD*	0.99	0.99
E. coli MG1655 (K-12) vs *E. coli* ECOR11 (R2)	*rfaD*	0.95	0.95
E. coli MG1655 (K-12) vs *E. coli* ECOR11 (R2)	*waaF*	0.99	1
E. coli MG1655 (K-12) vs *E. coli* ECOR11 (R2)	*waaC*	0.94	0.96
E. coli MG1655 (K-12) vs *E. coli* ECOR11 (R2)	*waaZ*	0.82	0.87
E. coli MG1655 (K-12) vs *E. coli* ECOR11 (R2)	*waaY*	0.92	0.96
E. coli MG1655 (K-12) vs *E. coli* ECOR11 (R2)	*waaR*	0.9	0.94
E. coli MG1655 (K-12) vs *E. coli* ECOR11 (R2)	*waaO*	0.88	0.93
E. coli MG1655 (K-12) vs *E. coli* ECOR11 (R2)	*waaB*	0.92	0.95
E. coli MG1655 (K-12) vs *E. coli* ECOR11 (R2)	*waaP*	0.91	0.94
E. coli MG1655 (K-12) vs *E. coli* ECOR11 (R2)	*waaG*	0.96	0.97
E. coli MG1655 (K-12) vs *E. coli* ECOR11 (R2)	*waaQ*	0.98	0.99
E. coli MG1655 (K-12) vs *E. coli* ECOR11 (R2)	*waaA*	1	1
E. coli MG1655 (K-12) vs *E. coli* ECOR11 (R2)	*coaD*	0.99	0.99
E. coli GF4-3 (R2/K-12) vs *E. coli* isolate EPEC E2348_69 variety 2 (K-12/R2)	*rfaD*	1	1
E. coli GF4-3 (R2/K-12) vs *E. coli* isolate EPEC E2348_69 variety 2 (K-12/R2)	*waaF*	0.99	0.99
E. coli GF4-3 (R2/K-12) vs *E. coli* isolate EPEC E2348_69 variety 2 (K-12/R2)	*waaC*	0.95	0.97
E. coli GF4-3 (R2/K-12) vs *E. coli* isolate EPEC E2348_69 variety 2 (K-12/R2)	*waaZ*	0.85	0.88
E. coli GF4-3 (R2/K-12) vs *E. coli* isolate EPEC E2348_69 variety 2 (K-12/R2)	*waaY*	0.97	0.97
E. coli GF4-3 (R2/K-12) vs *E. coli* isolate EPEC E2348_69 variety 2 (K-12/R2)	*waaR*	0.97	0.98
E. coli GF4-3 (R2/K-12) vs *E. coli* isolate EPEC E2348_69 variety 2 (K-12/R2)	*waaO*	0.95	0.98
E. coli GF4-3 (R2/K-12) vs *E. coli* isolate EPEC E2348_69 variety 2 (K-12/R2)	*waaB*	0.94	0.96
E. coli GF4-3 (R2/K-12) vs *E. coli* isolate EPEC E2348_69 variety 2 (K-12/R2)	*waaP*	0.92	0.95
E. coli GF4-3 (R2/K-12) vs *E. coli* isolate EPEC E2348_69 variety 2 (K-12/R2)	*waaG*	0.99	0.99

E. coli GF4-3 (R2/K-12) vs *E. coli* isolate EPEC E2348_69 variety 2 (K-12/R2)	*waaQ*	0.99	1
E. coli GF4-3 (R2/K-12) vs *E. coli* isolate EPEC E2348_69 variety 2 (K-12/R2)	*waaA*	1	1
E. coli GF4-3 (R2/K-12) vs *E. coli* isolate EPEC E2348_69 variety 2 (K-12/R2)	*coaD*	0.99	0.99
E. coli GF4-3 (R2/K-12) vs E. coli ECOR11 (R2)	*rfaD*	0.94	0.95
E. coli GF4-3 (R2/K-12) vs E. coli ECOR11 (R2)	*waaF*	1	1
E. coli GF4-3 (R2/K-12) vs E. coli ECOR11 (R2)	*waaC*	0.95	0.96
E. coli GF4-3 (R2/K-12) vs E. coli ECOR11 (R2)	*waaZ*	0.81	0.88
E. coli GF4-3 (R2/K-12) vs E. coli ECOR11 (R2)	*waaY*	0.91	0.95
E. coli GF4-3 (R2/K-12) vs E. coli ECOR11 (R2)	*waaR*	0.9	0.95
E. coli GF4-3 (R2/K-12) vs E. coli ECOR11 (R2)	*waaO*	0.85	0.91
E. coli GF4-3 (R2/K-12) vs E. coli ECOR11 (R2)	*waaB*	0.97	0.97
E. coli GF4-3 (R2/K-12) vs E. coli ECOR11 (R2)	*wabA*	0.99	0.99
E. coli GF4-3 (R2/K-12) vs E. coli ECOR11 (R2)	*waaP*	0.91	0.95
E. coli GF4-3 (R2/K-12) vs E. coli ECOR11 (R2)	*waaG*	0.89	0.93
E. coli GF4-3 (R2/K-12) vs E. coli ECOR11 (R2)	*waaQ*	0.71	0.8
E. coli GF4-3 (R2/K-12) vs E. coli ECOR11 (R2)	*waaA*	1	1
E. coli GF4-3 (R2/K-12) vs E. coli ECOR11 (R2)	*coaD*	1	1
E. coli isolate EPEC E2348_69 variety 2 (K-12/R2) vs *E. coli* ECOR11 (R2)	*rfaD*	0.95	0.95
E. coli isolate EPEC E2348_69 variety 2 (K-12/R2) vs *E. coli* ECOR11 (R2)	*waaF*	0.99	0.99
E. coli isolate EPEC E2348_69 variety 2 (K-12/R2) vs *E. coli* ECOR11 (R2)	*waaC*	0.97	0.97
E. coli isolate EPEC E2348_69 variety 2 (K-12/R2) vs *E. coli* ECOR11 (R2)	*waaL*	0.94	0.97
E. coli isolate EPEC E2348_69 variety 2 (K-12/R2) vs *E. coli* ECOR11 (R2)	*waaU*	0.91	0.93
E. coli isolate EPEC E2348_69 variety 2 (K-12/R2) vs *E. coli* ECOR11 (R2)	*waaZ*	0.86	0.91
E. coli isolate EPEC E2348_69 variety 2 (K-12/R2) vs *E. coli* ECOR11 (R2)	*waaY*	0.93	0.96

E. coli isolate EPEC E2348_69 variety 2 (K-12/R2) vs *E. coli* ECOR11 (R2)	*waaR*	0.89	0.94
E. coli isolate EPEC E2348_69 variety 2 (K-12/R2) vs *E. coli* ECOR11 (R2)	*waaO*	0.86	0.91
E. coli isolate EPEC E2348_69 variety 2 (K-12/R2) vs *E. coli* ECOR11 (R2)	*waaB*	0.92	0.95
E. coli isolate EPEC E2348_69 variety 2 (K-12/R2) vs *E. coli* ECOR11 (R2)	*waaP*	0.86	0.91
E. coli isolate EPEC E2348_69 variety 2 (K-12/R2) vs *E. coli* ECOR11 (R2)	*waaG*	0.88	0.92
E. coli isolate EPEC E2348_69 variety 2 (K-12/R2) vs *E. coli* ECOR11 (R2)	*waaQ*	0.71	0.8
E. coli isolate EPEC E2348_69 variety 2 (K-12/R2) vs *E. coli* ECOR11 (R2)	*waaA*	1	1
E. coli isolate EPEC E2348_69 variety 2 (K-12/R2) vs *E. coli* ECOR11 (R2)	*coaD*	0.99	0.99

References

1. **Gilchrist CLM, Chooi Y-H**. clinker & clustermap.js: automatic generation of gene cluster comparison figures. *Bioinformatics* 2021;37:2473–2475.
